# PI3K as Mediator of Apoptosis and Contractile Dysfunction in TGFβ_1_-Stimulated Cardiomyocytes

**DOI:** 10.3390/biology10070670

**Published:** 2021-07-16

**Authors:** Paulin Brosinsky, Julia Bornbaum, Björn Warga, Lisa Schulz, Klaus-Dieter Schlüter, Alessandra Ghigo, Emilio Hirsch, Rainer Schulz, Gerhild Euler, Jacqueline Heger

**Affiliations:** 1 Institute of Physiology, Justus-Liebig-University Giessen, 35392 Giessen, Germany; Paulin.Brosinsky@physiologie.med.uni-giessen.de (P.B.); Julia.Bornbaum@physiologie.med.uni-giessen.de (J.B.); B.Warga@umm.de (B.W.); Li.Schulz94@gmail.com (L.S.); Klaus-Dieter.Schlueter@physiologie.med.uni-giessen.de (K.-D.S.); Rainer.Schulz@physiologie.med.uni-giessen.de (R.S.); Gerhild.Euler@physiologie.med.uni-giessen.de (G.E.); 2 Department of Molecular Biotechnology and Health Sciences, Molecular Biotechnology Center, University of Torino, 10126 Torino, Italy; alessandra.ghigo@unito.it (A.G.); emilio.hirsch@unito.it (E.H.)

**Keywords:** TGFβ_1_, PI3K, cardiomyocytes, apoptosis, contractile function, SMAD, ALK5

## Abstract

**Simple Summary:**

TGFβ_1_ is a growth factor that plays a major role in the remodeling process of the heart by inducing cardiomyocytes dysfunction and apoptosis, as well as fibrosis, thereby restricting heart function. TGFβ_1_ mediates its effect via the TGFβ receptor I (ALK5) and the activation of SMAD transcription factors. But, TGFβ_1_ is also known as activator of phosphoinositide-3-kinase (PI3K) via the non-SMAD signaling pathway. The aim of this study was to investigate whether PI3K is also involved in TGFβ_1_–induced cardiomyocytes apoptosis and contractile dysfunction. Pharmacological inhibition of PI3K with Ly294002 reduced TGFβ-induced apoptosis and reduced cell shortening. Inhibition of the PI3Kγ isoform also abolished the TGFβ effect on apoptosis and cell shortening. These data support a role for a PI3K and ALK5/SMAD pathway in TGFβ_1_-induced apoptosis and impaired cell shortening, which in part appears to be PI3Kγ-dependent.

**Abstract:**

Background: TGFβ_1_ is a growth factor that plays a major role in the remodeling process of the heart by inducing cardiomyocyte dysfunction and apoptosis, as well as fibrosis thereby restricting heart function. TGFβ_1_ mediates its effect via the TGFβ receptor I (ALK5) and the activation of SMAD transcription factors, but TGFβ_1_ is also known as activator of phosphoinositide-3-kinase (PI3K) via the non-SMAD signaling pathway. The aim of this study was to investigate whether PI3K is also involved in TGFβ_1_–induced cardiomyocytes apoptosis and contractile dysfunction. Methods and Results: Incubation of isolated ventricular cardiomyocytes with TGFβ_1_ resulted in impaired contractile function. Pre-incubation of cells with the PI3K inhibitor Ly294002 or the ALK5 inhibitor SB431542 attenuated the decreased cell shortening in TGFβ_1_–stimulated cells. Additionally, TGFβ-induced apoptosis was significantly reduced by the PI3K inhibitor Ly294002. Administration of a PI3Kγ-specific inhibitor AS605240 abolished the TGFβ effect on apoptosis and cell shortening. This was also confirmed in cardiomyocytes from PI3Kγ KO mice. Induction of SMAD binding activity and the TGFβ target gene collagen 1 could be blocked by the PI3K inhibitor Ly294002, but not by the specific PI3Kγ inhibitor AS605240. Conclusions: TGFβ_1_-induced SMAD activation, cardiomyocyte apoptosis, and impaired cell shortening are mediated via both, the ALK5 receptor and PI3K, in adult cardiomyocytes. PI3Kγ specifically contributes to apoptosis induction and impairment of contractile function independent of SMAD signaling.

## 1. Introduction

The transition of compensated hypertrophy to heart failure is accompanied by an increased expression of transforming growth factor β (TGFβ) [1,2] that contributes to cardiomyocyte apoptosis [3], enhanced β-adrenergic signaling [4] and contractile dysfunction [5,6,7], since overexpression of TGFβ in transgenic mice led to a moderate increase of myocardial β-adrenoceptor density and increased prohypertrophic signaling [1].

TGFβ mediates its signaling by binding to a heteromeric serine/threonine kinase receptor complex in the membrane [8,9], which is composed of the constitutive active type II TGFβ receptor that, upon binding of TGFβ, phosphorylates type I TGFβ receptor (ALK5) [10]. This results in activation of the canonical small other against decapentaplegic (SMAD) signaling cascade by phosphorylation of SMAD2/SMAD3 and binding of co-SMAD4 that translocates into the nucleus [3,11]. Besides activation of ALK5, TGFβ receptor II also activates a non-SMAD signaling cascade including p38 mitogen-activated protein (MAP) kinase [12], extracellular-signal-related kinase (ERK) [13,14], protein phosphatase 2A (PP2a) [15], c-Jun N-terminal kinase (JNK) [16] and PI3K [17]. The TGFβ receptor II is constitutively associated with the regulatory subunit of PI3K, p85, [18], whereas association of p85 with TGFβ receptor I needs activation with TGFβ [19]. Activation of PI3K can be beneficial as well as detrimental for the heart [20]. This may depend on the stimulus used, the involved PI3K isoform and the cell type that is affected.

PI3K belongs to a family of lipid kinases and can be divided into three classes [21,22,23]. Class I PI3Ks are best characterized and can be subdivided in PI3K α, β, γ, and δ. They function as heterodimers composed of the catalytic subunit p110 (α, β, γ, δ) and an adaptor subunit (p84/87 or p101 for PI3Kγ, as well as p85 for PI3Kα, β and δ) that mediates binding to receptors and activation of enzymes [24]. PI3Kα and PI3Kβ are ubiquitously expressed [25]. In contrast, PI3Kγ is essentially expressed in cardiomyocytes, hematopoietic cells, and endothelial cells [26]. In the heart, PI3Kα mainly seems to be involved in the control of physiologic cardiac hypertrophy [27,28,29], thereby acting in a cardioprotective manner [30,31]. PI3Kγ is a stress kinase activated by various cardiac conditions [32]. PI3Kγ is also a scaffold protein influencing the cAMP compartmentalization [33] and is implicated in the fine tuning of cardiac contractile function [27,33,34,35]. Here the PI3Kγ-dependent switch of cyclic adenosine monophosphate (cAMP) compartmentalization affects multiple β_2_–AR/cAMP microdomains via protein kinase A (PKA)-mediated activation of distinct phosphodiesterases (PDEs) [33]. This mechanism is relevant for regulating cardiac function and and once out of balance appears to be involved in the development of heart failure.

We now wanted to analyze to what extent TGFβ stimulation affects adult cardiomyocyte function and apoptosis through the classical ALK5 or the PI3K pathway and to what extent the PI3Kγ isoform is involved in the latter.

## 2. Materials and Methods

### 2.1. Animals

Female and male wild type C57BL/6J mice (Janvier SAS, Le Genest Saint Isle, France), PI3Kγ knock-out mice (kindly provided by the working group of Professor Emilio Hirsch, Department of Molecular Biotechnology and Health Sciences, Center for Molecular Biotechnology, Torino, Italy) and wild type male Wistar rats (Janvier SAS) were used for preparation of isolation of cardiomyocytes. This study was approved by the institutional animal care committee of the Justus-Liebig-University Giessen and was registered under the number 668_M and 644_M.

### 2.2. Materials

Medium 199 was obtained from Boehringer (Mannheim, Germany) and fetal calf serum from PAA (Linz, Austria). Crude collagenase was bought from Biochrom (Berlin, Germany), TGFβ_1_ and Ly294002 from Sigma (Merck Bioscience, Darmstadt, Germany), laminin from Roche (Mannheim, Germany), oligonucleotides were from Invitrogen (Karlsruhe, Germany), SB431542 from Cayman (Biomol, Hamburg, Germany) and AS605240 from Selleckchem (Biozol, Eching, Germany).

### 2.3. Isolation of Murine Cardiomyocytes

Mice were anaesthetized by an inhalation of 5% isoflurane followed by the euthanasia with the cervical dislocation. The hearts were extracted and rinsed with 4 °C cold 0.9% NaCl. Thereafter, the hearts were digested with retrograde perfusion in a Langendorff apparatus for 25 min containing collagenase and calcium-free buffer (in mmol/L: 10 glucose monohydrate D^+^, 25 HEPES, 2.5 KCl, 1.2 KH_2_PO_4_, 1.2 MgSO_4_ × 7 H_2_O, 110 NaCl) with a pH of 7.4 at 37 °C. Subsequently, the tissue was minced and incubated for another five minutes in the recirculating buffer. The suspension was filtered and the cardiomyocytes were separated from the other cells by centrifugation. To reconstruct the physiological calcium concentration the material was resuspended with an step-wise increase of calcium up to 1 mM followed by resuspension in the culture medium (in mmol/L: 2.5 CaCl_2_-dihydrate, 5 glucose, 10 HEPES, 4.7 KCl, 1.2 KH_2_PO_4_, 0.8 MgSO_4_, 118 NaCl and 1.9 Na-pyruvate). Finally, the cardiomyocytes were plated on laminin-coated (5 µg/mL) culture dishes and incubated at 37 °C (5.5% CO_2_, 95% humidity). After 1 h, the cells were washed with fresh culture medium and could be then stimulated. The cardiomyocytes were treated with TGFβ_1_. Times of stimulation are given for the respective experiments in the figure legends. TGFβ_1_ concentrations will be given in the figure legends. Untreated samples served as a control.

### 2.4. Isolation of Rat Cardiomyocytes

The isolation of ventricular cardiomyocytes from 200 to 250 g male Wistar rats were performed similar to the isolation of murine cardiomyocytes. The cells were suspended in culture medium and subsequently plated onto preincubated culture dishes (4% fetal calf serum in medium 199, overnight). Two hours later, the cells were washed twice with fresh CCT medium (modified medium 199 including Earle’s salts, 2 mM l-carnitine, 5 mmol/L taurine, 100,000 IU/L penicillin, 100 mg/L streptomycin and 10 μmol/L cytosine-β-d-arabinofuranoside (pH 7.4). On average, about 90% of the cultures were quiescent rod-shaped cells. Subsequent to the cell washing, they were treated with 1 ng TGFβ_1_. Untreated samples served as controls.

### 2.5. Determination of Cell Function

Cell contraction behavior was analysed at room temperature by using a cell-edge-detection system. The cultured cardiomyocytes were stimulated by using two AgCl electrodes with biphasic electrical stimuli constituted out of two equal but opposite rectangular 50 V stimuli of 0.5 ms duration. Each cell was stimulated with a voltage of 2 Hz and was measured four times. To calculate the cell shortening of a given cell, the mean of these measurements was used. In addition, cell lengths could be analysed by using a line camera, recording at 500 Hz. The resulting data are presented as cell shortening normalized to diastolic cell length (dL/L (%)).

### 2.6. Electrophoretic Mobility Shift Assay (EMSA)

For the extraction of nuclear extracts, isolated cardiomyocytes were homogenized in swelling buffer (10 mM Tris–HCl, pH 7.9, 1 mM MgCl_2_, 10 mM KCl, 1 mM DTT). Thereafter, the homogenates were placed on ice for one hour and nuclei were pelleted by centrifugation at 900 rpm for ten minutes. Supernatants were used for Western blots. Pellets were homogenized in 10 mM Tris–HCl, pH 7.9, 300 mM sucrose, 1mM DTT, 1.5 mM MgCl_2_, 0.3% triton X-100 and another time centrifuged as above. These pellets were resuspended in storage buffer (10 mM HEPES, pH 7.5, 50 mM KCl, 300 mM NaCl, 1 mM EDTA, 1 mM DTT, 1 mM PMSF, 20% glycerol) on ice for 30 min and centrifuged at 13,000 rpm, 4 °C, for five minutes. For electrophoretic mobility shift assays (EMSAs) the resulting supernatants were taken. Complementary sequences of SBE (SMAD binding elements) oligonucleotides were hybridized. 5′-GTACAT TGTCAGTCTAGACATACT-3′ was the sequence for binding of SMAD transcription factors [3]. In the presence of Cy3-dCTP, oligonucleotides were incubated with terminal transferase. Using gel filtration. Unincorporated nucleotides were parted from fluorescence-labeled oligonucleotides. Ten μL nuclear extracts were incubated with labeled oligonucleotides in the presence of one μg poly (dIdC) at 30 °C for 30 min. The samples were run on 4% native polyacrylamide gels. Complete gels were exposed on fluorescence imager (BioRad, Hercules, CA, USA).

### 2.7. Western Blot Analysis

To lyse the supernatans of the homogenized cardiomyocytes, lysis buffer [(Cell Signaling, Beverly, MA, USA) containing Tris (pH 7.5) 20 mmol/L, NaCl 150 mmol/L, EDTA 1 mmol/L, sodium pyrophosphate 2.5 mmol/L, EGTA 1 mmol/L, Na_3_VO_4_ 1 mmol/L, β-glycerophosphate 1 mmol/L, Triton X-100 1%, leupeptin 1 µg/mL, supplemented with 1× Complete Protease Inhibitor Cocktail (Roche, Basel, Switzerland)] was used followed by a 10 min centriufation step at 13,000× *g*. By using DC Protein Assay kit (BioRad) protein concentration was determined. 50 µg proteins were electrophoretically separated on 10% Bis-Tris gels. After transferring the proteins onto a nitrocellulose membrane, they were blocked with 5% skim milk powder in TBS/1% Tween for one hour at room temperature. Afterwards the membranes were incubated at 4 °C overnight with primary antibody pSMAD2 (Ser465/467) (Cell Signaling Technology, Frankfurt, Germany, #3108S), SMAD2 (Cell Signaling Technology, #3103S) or Vinculin (Sigma, Taufkirchen, Germany, V9131) at 4 °C. HRP-conjugated goat anti-rabbit IgG or goat-anti mouse IgG were used as secondary antibody (Cell Signaling Technology) for one hour at room temperatur. Immuno-reactive bands were detected using the SuperSignal West Femto Maximum Sensitivity Substrate (Pierce, Rockford, IL, USA). Protein bands were quantified with Quantity One software Version 4.6.9 (Bio-Rad Laboratories GmbH, Feldkirchen, Germany).

### 2.8. Real-Time RT-PCR

According to the instructions by the manufacturer, total RNA of cardiomyocytes was extracted with Trizol (Invitrogen, Hercules, CA, USA). Reverse transcription was done for one hour at 37 °C in a final volume of 10 µL using 1 µg of RNA, eight units of RNasin, 1 mol/L dNTPs, 100 ng of oligo (dT)_15_ and 60 units of Moloney murine leukemia virus reverse transcriptase. Aliquots (1.5 µL) of the synthesized cDNA were used for PCR in a final volume of 10 µL including primer pairs at 1.5 µmol/L, 1.5 mol/L MgCl_2_, 0.4 mol/L dNTPs, and 1 unit of *Taq* polymerase. For each tested gene, annealing temperature, and the number of cycles resulting in a linear amplification range were tested. RT-PCR was run in an automated thermal cycler. SYBR Green fluorescence was used for quantification and detected with the Bio-Rad detection system (Bio-Rad). The calculations of the results were done according to the 2^−ΔΔCt^ methods as described [36]. Determination of the “most stable” housekeeping genes in real-time RT-PCR was performed by using repeated pair-wise correlation analysis of GAPDH 18sRNA, B2M, and HPRT [37] as listed in Table 1.

### 2.9. Caspase 3/7 Assay

The kit “Caspase-Glo 3/7” (Promega, Mannheim, Germany) was used for the measurement of caspase activity as recommended by the company. In brief, cardiomyocytes were stimulated with TGFβ_1_ for six hours. Thereafter, a caspase-3/7 pro-luminescent substrate was added to the cells. This led to cell lysis and caspase cleavage of the substrate. Using a spectrophotometer the generation of a “glow-type” luminescent signal could be measured. The values obtained were normalised to the amount of protein per well.

### 2.10. Statistical Analysis

The SPSS programme (IBM SPSS Statistics 27, IBM Deutschland GmbH, Ehningen, Germany) was used for statistical data analysis. Results are expressed as mean ± standard error of the mean (SEM) as indicated in the figure legends. A one-way ANOVA was applied followed by Student-Newman-Keuls post hoc test, and the Mann-Whitney-U test to further evaluate differences between two means. Differences among multiple groups were evaluated using a two-way analysis of variance (ANOVA) for the two factors genotype (WT versus PI3Kγ knock-out) and treatment (with/without TGFβ_1_), followed by Tukey’s post hoc test. Values of *p* < 0.05 were marked to be statistically significant.

## 3. Results

### 3.1. TGFβ_1_–Induced Apoptosis Depends on PI3K

**Figure 1:**
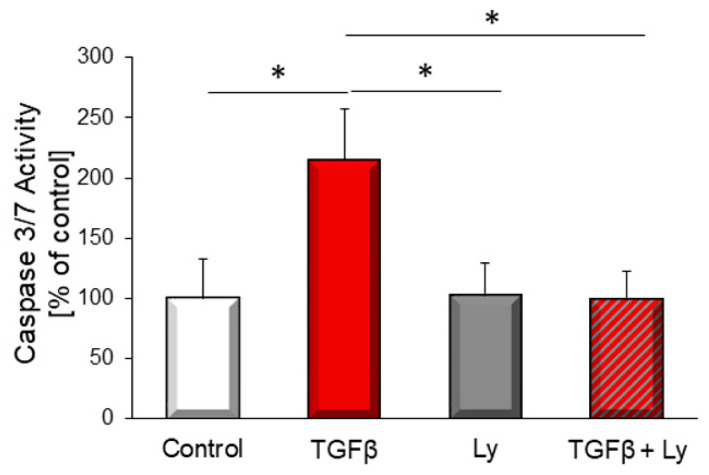
TGFβ_1_–induced apoptosis was determined with and without addition of the PI3K inhibitor Ly294002 (10 µM) by caspase 3/7 activation after six hours stimulation with 1 ng/mL TGFβ_1_. Data are shown as means ± SEM of 11 different rat cardiomyocytes preparations. Significance is indicated by * *p* < 0.05.

To study the impact of PI3K on TGFβ_1_–induced apoptosis, adult rat cardiomyocytes were treated with TGFβ_1_ and the PI3K inhibitor Ly294002. Increase of caspase 3/7 activation by TGFβ_1_ was reduced by the administration of Ly294002 (Figure 1).

### 3.2. Involvement of PI3K in TGFβ_1_-Dependent SMAD Binding Activity and TGFβ Target Genes

**Figure 2:**
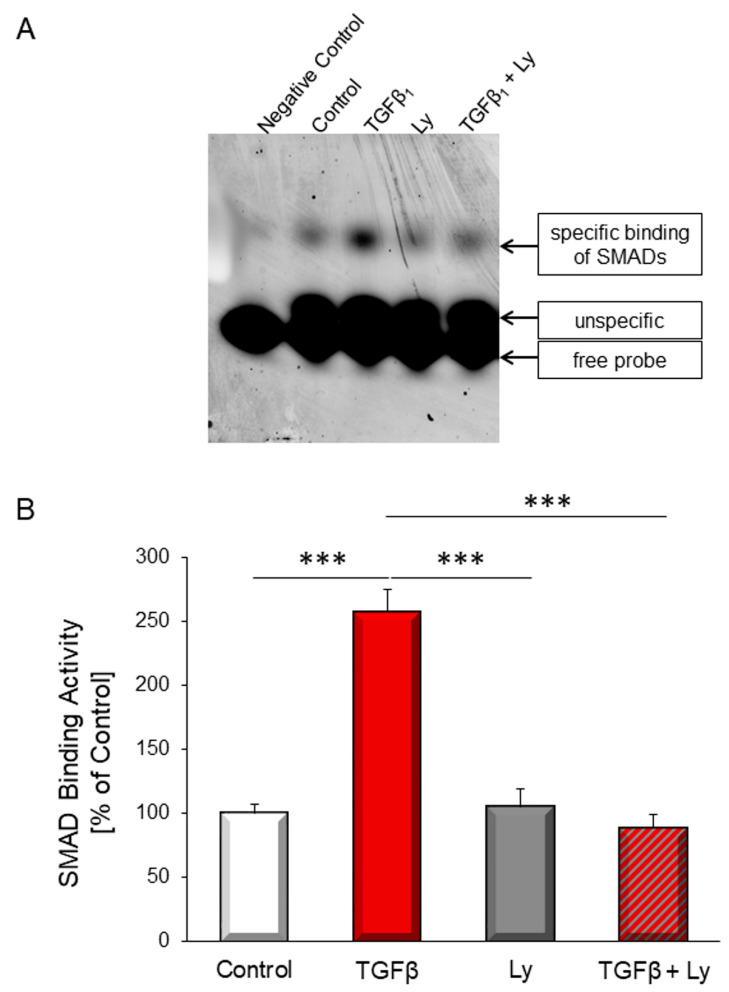
TGFβ_1_–induced SMAD binding activity was determined with and without addition of PI3K inhibitor Ly294002 (10 µM) after two hours stimulation with 1 ng/mL TGFβ_1_. (**A**) Shows a representative EMSA and (**B**) the quantitative analysis. Data are shown as means ± SEM of four different rat cardiomyocytes preparations. Significance is indicated by *** *p* < 0.001.

TGFβ_1_–induced apoptosis is mediated via SMAD transcription factors. To evaluate whether PI3K is involved in enhanced SMAD binding activity under TGFβ stimulation, electromobility shift assays were performed. Increase in SMAD binding activity could be abolished by inhibition of PI3K with Ly294002 (Figure 2).

**Figure 3:**
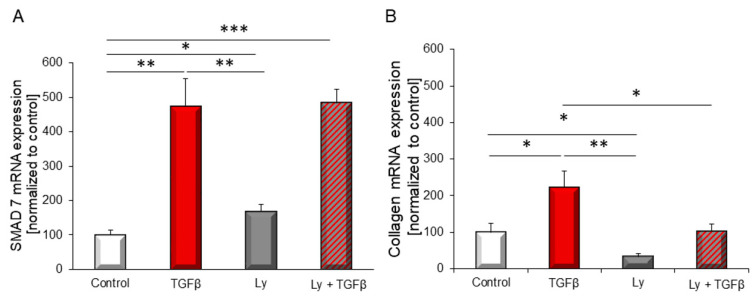
mRNA expression of TGFβ_1_ target genes (**A**) *SMAD7*, (**B**) *collagen 1* was determined with and without addition of PI3K inhibitor Ly294002 (10 µM) after 24 h stimulation with 1 ng/mL TGFβ_1_. Gene expression was normalized to bestkeeper (18sRNA + B2M for *SMAD7*, B2M + GAPDH for *collagen 1*). Data are shown as means ± SEM of seven different rat cardiomyocytes preparations. Significance is indicated by * *p* < 0.05, ** *p* < 0.01 and *** *p* < 0.001.

Analysis of two TGFβ target genes revealed an increase in expression upon TGFβ stimulation. Whereas SMAD7 expression was not affected, collagen 1 mRNA expression was down-regulated by the PI3K inhibitor Ly294002 (Figure 3).

### 3.3. PI3K Affected TGFβ_1_-Dependent Depression of Contractile Function

**Figure 4:**
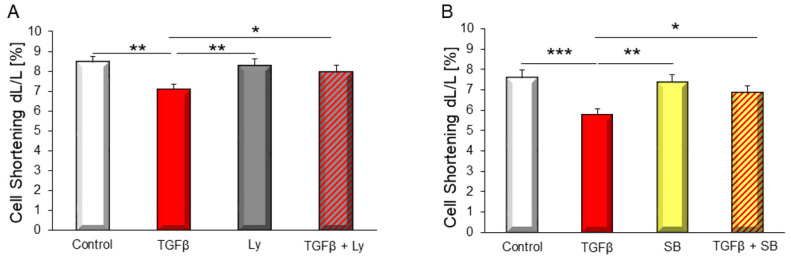
Cell shortening of isolated cardiomyocytes was determined after stimulation with 1 ng/mL TGFβ_1_ with and without addition of PI3K inhibitor Ly294002 (Ly, 1 µM) (**A**) or ALK5 inhibitor SB431542 (SB, 1 µM) (**B**). Data are shown as means ± SEM of 71 to 87 cells of four to seven different rat cardiomyocytes preparations. Significance is indicated by * *p* < 0.05, ** *p* < 0.01 *** and *p* < 0.001.

Isolated cardiomyocytes of adult rats responded with a decrease in cell shortening upon TGFβ_1_ stimulation. Pre-incubation of the cells with Ly294002 abolished this effect (Figure 4A). In addition, pre-incubation of cardiomyocytes with TGFβ receptor I (ALK5) inhibitor SB431542, similarly inhibited the impairment of cardiomyocytes contractile function by TGFβ (Figure 4B) demonstrating that both PI3K and SMADs mediate contractile dysfunction of cardiomyocytes under TGFβ stimulation.

### 3.4. PI3Kγ Is Involved in TGFβ_1_-Dependent Depression of Contractile Function and in TGFβ-Dependent Apoptosis Induction

**Figure 5:**
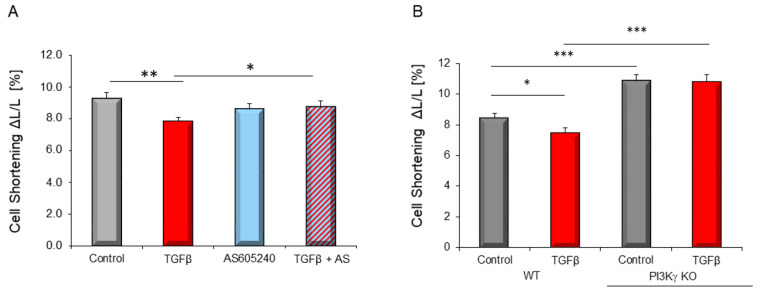
(**A**) Contractile function of isolated cardiomyocytes of adult rats. Cardiomyocytes were incubated with TGFβ_1_ (1 ng/mL) for 24 h, with and without addition of PI3Kγ inhibitor AS605240 (100 nM), afterwards stimulated with 2 Hz and contractile parameters were measured. Data are mean ± SEM of 54 cells of six independent cardiomyocytes preparations. (**B**) Contractile function of isolated cardiomyocytes of PI3Kγ KO and WT mice. Cardiomyocytes were incubated with TGFβ_1_ (10 ng/mL) for 24 h, afterwards stimulated with 2 Hz and contractile parameters were measured. Data are mean ± SEM of 6–10 different cell dishes with 54 to 90 cells. dL/L: shortening amplitude normalized to diastolic cell length (amplitude × 100/L Diast). Significance is indicated by * *p* < 0.05, ** *p* < 0.01 and *** *p* < 0.001. Two-way ANOVA: WT versus PI3Kγ KO *p* < 0.0001.

Since PI3Kγ is highly expressed in cardiomyocytes, we now analyzed, if this specific isoform contributes to TGFβ-induced contractile dysfunction. Pharmacological inhibition of PI3Kγ by AS605240 did not affect baseline cardiomyocytes shortening but led to a reduction of TGFβ_1_-dependent depression of cardiomyocytes shortening (Figure 5A). This was validated in cardiomyocytes from PI3Kγ KO mice. Whereas WT cardiomyocytes responded to TGFβ stimulation with a decrease in cell shortening, PI3Kγ cardiomyocytes were not affected by TGFβ (Figure 5B). This demonstrates that PI3Kγ is involved in the impairment of contractile function under TGFβ stimulation.

**Figure 6:**
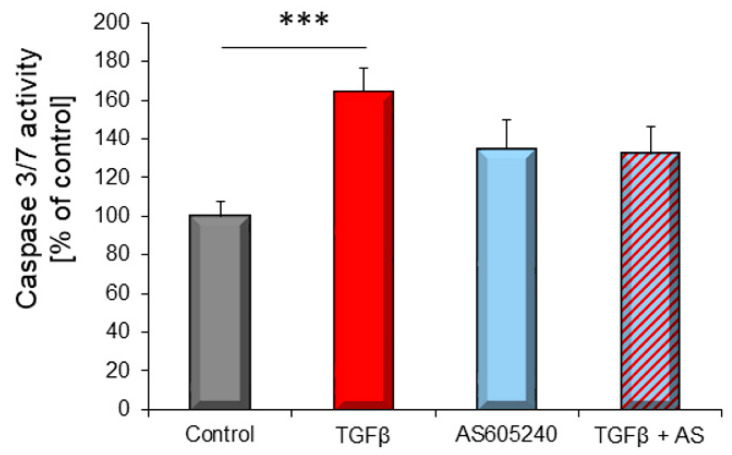
Cardiomyocytes of adult rats were stimulated with 1 ng/mL TGFβ_1_ for 6 h, with and without addition of PI3Kγ inhibitor AS605240 (1 µM). Quantitative determination of caspase 3/7 activation (*n* = 14–16 independent cardiomyocytes preparations). Data are shown as means ± SEM. Significance is indicated by *** *p* < 0.001.

Since PI3Kγ influences cardiomyocyte function, we wanted to know whether this isoform also has an influence on apoptosis. Pharmacological inhibition of PI3Kγ by AS605240 blocked TGFβ_1_–induced caspase 3/7 activation (Figure 6).

### 3.5. PI3Kγ Action Is Independent of SMAD Activation

**Figure 7:**
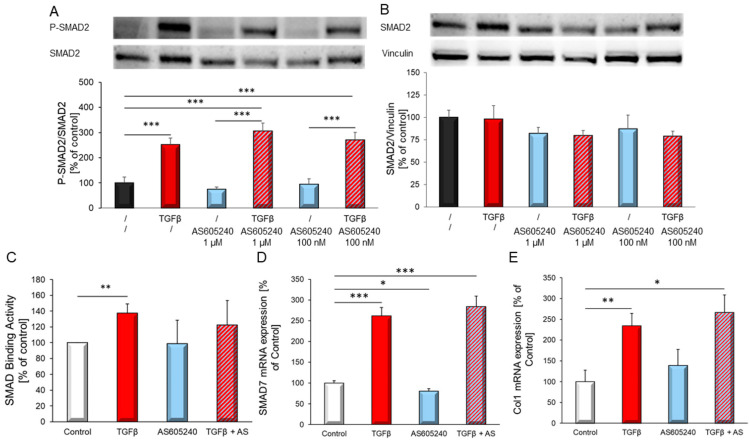
TGFβ_1_-induced pSMAD2 (Ser465/467). (**A**) and SMAD2 (**B**) expression was determined with and without addition of the PI3Kγ inhibitor AS605240 (100 nM, 1 µM). Representative Western blot and quantitative analysis, (*n* = 7 different cardiomyocytes preparations). (**C**) TGFβ_1_-induced SMAD binding activity was determined with and without addition of the PI3Kγ inhibitor AS605240 (100 nM) (*n* = 17 different cardiomyocytes preparations). mRNA expression of TGFβ_1_ target genes (**D**) *SMAD7* (**E**) *collagen 1* was determined with and without addition of the PI3Kγ inhibitor AS605240 (100 nM) after 24 h TGFβ_1_ stimulation (*n* = 6 different rat cardiomyocytes preparations). Gene expression was normalized to the most stable housekeeping gene “bestkeeper” (GAPDH, HPRT, 18sRNA). Data are shown as means ± SEM. Significance is indicated by * *p* < 0.05, ** *p* < 0.01 and *** *p* < 0.001.

Further, we analysed whether the influence of the TGFβ_1_-dependent effects of PI3Kγ were linked to the canonical TGFβ signalling pathway. SMAD activation demonstrated by phosphorylation of SMAD2 revealed no inhibition of TGFβ-induced SMAD2 activation by the use of the PI3Kγ-specific inhibitor AS605240. (Figure 7A,B). Addition of the PI3Kγ inhibitor AS605240 did not block SMAD binding activity (Figure 7C). Also, expression of the TGFβ target genes SMAD7 and collagen 1 were not affected by inhibition of PI3Kγ (Figure 7D,E).

## 4. Discussion

We have identified PI3K as a further signalling molecule involved in TGFβ-mediated decrease in cell shortening as well as apoptosis in cardiomyocytes. That TGFβ is capable of decreasing cell shortening was demonstrated in adult rat cardiomyocytes stimulated with AngII [6], which induces activation and release of TGFβ [38]. Addition of neutralizing TGFβ antibodies into the culture medium of AngII-stimulated cardiomyocytes improved their function [6]. Banerjee et al. [39] also demonstrated that TGFβ decreased contractile function in embryonic cardiomyocytes of mice in a SMAD-dependent manner. Isolated cardiomyocytes of TGFβ-overexpressing mice had an attenuated cell shortening [7], which was accompanied by decreased mitochondrial energy metabolism due to increased expression of mitochondrial uncoupling proteins (UCP). However, none of the above studies proposed an involvement of PI3K in TGFβ-mediated contractile dysfunction.

Both beneficial and detrimental roles in the regulation of cardiomyocyte contractility have already been shown for PI3K. Enhancement of PI3Kα or reduction of PI3Kγ as a therapeutic approach in heart disease has been suggested [40]. Accordingly, overexpression of cardiac-specific PI3Kα resulted in increased contractility in mice [41], whereas cardiac-specific ablation of p110α—the catalytic subunit of PI3Kα—in adult mice caused significant reductions in voltage-dependent L-type Ca^2+^ channel density, Ca^2+^ transients, and cardiomyocyte contraction that resulted in compromised cardiac contractility [42]. In contrast, targeted deletion of PI3Kγ significantly increased contractility [35,43] and enhanced cardiac function as assessed by increased fractional shortening [27,35]. Additionally, mice expressing a kinase-dead PI3Kγ (PI3K^KD^) displayed protection from maladaptive remodeling and cardiac dysfunction up to 16 weeks after aortic constriction [44]. Also in the present study, supporting previous findings, PI3Kγ KO improved cardiomyocytes shortening under basal conditions.

In contrast to the PI3Kγ KO cardiomyocytes, pharmacological PI3Kγ inhibition itself had no effect on the cardiomyocytes function of WT mice. However, the inhibition of PI3Kγ with AS605240 could reverse TGFβ-induced depression of cell shortening as seen also in PI3Kγ KO cardiomyocytes. Cardiomyocytes from PI3Kγ KO mice already have higher cell shortening due to cAMP elevation than WT mice already at baseline [34], and therefore TGFβ_1_ probably could not influence contractility in PI3Kγ KO cardiomyocytes. Our findings on differences in contractility depending on the complete KO of PI3Kγ or inhibition of PI3Kγ activity by AS605240 has been seen comparing PI3Kγ^−/−^ to PI3Kγ^KD^mice. In contrast to PI3Kγ KO mice, the loss of kinase activity of PI3Kγ^KD^ did not lead to a change in contractility [34].

The TGFβ and also the PI3K signalling pathway are important for the organism to control various cell responses, such as apoptosis. Many studies suggest that these two pathways may counteract each other, especially in cell survival where PI3K has a protective role via activation of AKT (for a review see Kandel and Hay [45]. Although PI3K signaling usually improves survival in combination with SMAD signaling this effect is reversed. The decisive factor seems to be the extent to which SMAD or AKT is in the predominance [46,47]. There is also evidence that both pathways work in the same direction, for example, for progression of tumour cells [48]. In addition to SMAD activation [3], we now demonstrate that PI3K is also necessary for TGFβ-induced apoptosis. This is supported by Bakin et al. [49], who showed an interaction between PI3K and TGFβ. In epithelial cells, inhibition of PI3K led to blockage of TGFβ-induced SMAD2 activation, which thereby regulated signaling molecules of TGFβ-induced apoptosis. In addition, Conery et al. [47] identified a crosstalk between the TGFβ and PI3K pathways in different cell lines that led to apoptosis. This study showed that with an increase in the amount of SMAD3 translocating into the nucleus, apoptosis induction was enhanced. We also could decrease SMAD translocation by inhibition of PI3K demonstrated by EMSA. This is in line with other studies showing PI3K/SMAD signaling being harmful for the heart [50,51].

Recently, Aki et al. [52] elucidated the crucial role of PI3K for TGFβ signaling in vascular endothelial cells. PI3Kα knockdown nearly completely abolished TGFβ_1_-induced phosphorylation and nuclear translocation of SMAD2/3—the canonical transcription factors downstream of TGFβ. Our study now identified PI3K as another down-stream signaling molecule of TGFβ-induced apoptosis in cardiomyocytes with PI3Kγ being the important isoform. Other studies confirm that PI3K modulates the SMAD signalling pathway and thus apoptosis. In the present study we did not see an influence of PI3Kγ on SMADS, although PI3Kγ did influence apoptosis. This suggests that PI3Kγ acts independently of SMADs.

SMAD transcription factors are the canonical mediators of TGFβ signaling, where SMAD2 and SMAD3 are phosphorylated upon receptor activation and SMAD7 acts as an inhibitory SMAD. Whereas SMAD7 transcription could be abolished by SB431542 [3], the inhibition of PI3K had no effect on SMAD7 transcription. Our findings fit quite well with the studies by Edlund et al. [53], here pretreatment with the PI3K inhibitor LY294002 did not abolish the Smad7-induced activation of Cdc42 in human prostate cancer cell line (PC-3U). They stated that Smad7 has probably different roles in addition to its inhibitory function in TGFβ signaling which suggests that there is a parallel signaling pathway. In contrast to SMAD7, the TGFβ-induced upregulation of collagen 1 expression could be blocked by inhibition of PI3K. This could also be measured in human mesangial cells, where the PI3K inhibitor LY294002 decreased TGFβ-stimulated Col1 mRNA expression by blocking induction of COL1A2 promoter activity [54]. This supports the notion that the TGFβ-induced increase in collagen 1 expression is PI3K-dependent. However, in the present study, PI3Kγ had no effect on collagen 1 expression, which was also supported by the fact that neither SMAD translocation nor SMAD2 phosphorylation could be inhibited. TGFβ-induced upregulation of collagen-1 expression may be mediated via another PI3K isoform that has not yet been identified.

## 5. Conclusions

Our data support a role for a PI3K and ALK5/SMAD pathway in TGFβ_1_-induced apoptosis and impaired cell shortening. More specifically, the influence on apoptosis and contractile dysfunction of TGFβ_1_ stimulation in part appears to be PI3Kγ-dependent.

## Figures and Tables

**Table 1:** Primer sequences used for real-time RT-PCR.

Gene	Accession Numbers	Forward Primer 5′→3′	Reverse Primer 5′→3′
*B2M*	NM_012512	GCCGTCGTGCTTGCCATTC	CTG AGG TGG GTG GAA CTG AGA C
*18sRNA*		Qiagen QT00199374	
*HPRT*	NM_012583	CCA GCG TCG TGA TTA GTG AT	CAA GTC TTT CAG TCC TGT CC
*GAPDH*	NM_017008	TCCATGCCATCACTGCCACTC	TGACCTTGCCCACAGCCTTG
*SMAD7*	AF042499	AGAGGCTGTGTTGCTGTG	CATCGGGTATCTGGAGTAAGG
*Collagen1*	NM_053304	GCG AAC AAG GTG ACA GAG	CCA GGA GAA CCA GCA GAG

## Data Availability

The data presented in this study are available on request from the corresponding author.
